# Green Chemistry for Crosslinking Biopolymers: Recent Advances in Riboflavin-Mediated Photochemistry

**DOI:** 10.3390/ma16031218

**Published:** 2023-01-31

**Authors:** Yoon Bok Lee, Saebin Lim, Yerin Lee, Chan Ho Park, Hyun Jong Lee

**Affiliations:** Department of Chemical and Biological Engineering, Gachon University, 1342 Seongnam-daero, Seongnam-si 13120, Gyeonggi-do, Republic of Korea

**Keywords:** riboflavin, riboflavin phosphate, visible light, photochemistry, thiol-ene reaction, furfuryl group

## Abstract

Riboflavin (RF), which is also known as vitamin B2, is a water-soluble vitamin. RF is a nontoxic and biocompatible natural substance. It absorbs light (at wavelengths of 380 and 450 nm) in the presence of oxygen to form reactive singlet oxygen (^1^O_2_). The generated singlet oxygen acts as a photoinitiator to induce the oxidation of biomolecules, such as amino acids, proteins, and nucleotides, or to initiate chemical reactions, such as the thiol-ene reaction and crosslinking of tyramine and furfuryl groups. In this review, we focus on the chemical mechanism and utilization of the photochemistry of RF, such as protein crosslinking and hydrogel formation. Currently, the crosslinking method using RF as a photoinitiator is actively employed in ophthalmic clinics. However, a significant broadening is expected in its range of applications, such as in tissue engineering and drug delivery.

## 1. Introduction

Biopolymers are nature-derived polymer materials that are produced by living organisms. Most natural biopolymers are biocompatible, biodegradable, naturally abundant, and sustainable [1,2]. Certain synthetic polymers developed for biomedical applications also exhibit biocompatibility or biodegradability, and chemical modifications of these polymers are more straightforward than those of biopolymers [3]. However, the increased utilization of biopolymers derived from natural sources is beneficial for environmental sustainability, as it reduces synthetic polymer production or promotes the usage of discarded biopolymers.

In the biomedical field, biopolymers are applied in wound healing, tissue engineering, drug delivery, and cosmetics [4,5]. Natural biopolymers have suitable biological properties for application in the biomedical field; however, they have limitations in their original state. The poor mechanical properties of biopolymers, such as low strength and rapid decomposition, must be improved. The crosslinking of biopolymers is a method for improving their mechanical properties. Crosslinking increases the molecular weight of biopolymers or forms an intermolecular network [6].

Biopolymers can be crosslinked via enzymatic reactions, light irradiation, and temperature and pH variations [7]. Although various stimuli can induce crosslinking, the light-mediated photochemistry-based crosslinking of biopolymers have recently gained significant research attention [8]. Crosslinking using light is more beneficial for spatiotemporal control than that using other stimuli [9], as it is challenging to swiftly control localized temperature changes, pH, and enzyme concentration [8]. However, light has a unique property: it can be triggered at a precise time and location. In addition, temperature, pH, and enzymes are internal parameters that maintain homeostasis in the body; however, light is an external trigger that is not controlled in the body. Other external triggers include ultrasound, magnetic fields, electric fields, and exogenous chemicals; however, light can induce rapid changes in local areas [8].

The light-induced chemical reactions require a photoinitiator. This review covers photochemical reactions that use riboflavin (RF) or riboflavin phosphate (RFP) as photoinitiators. The natural substances RF and RFP, which act as photoinitiators by absorbing light in the UV and blue light spectrums, are promising because of their excellent biocompatibility. We focus on cases using blue light with a wavelength of approximately 450 nm, which is within the visible light region, as a light source. Using current studies on RF-mediated photochemistry, we assess the future potential of this technique.

## 2. Visible Light-Induced Photochemistry

### 2.1. Visible Light-Induced Photoinitiators

Ultraviolet (UV) irradiation is frequently employed as an initiator in photochemical reactions. However, visible light is considered more biocompatible than UV light, as UV irradiation can damage cells and affect their viability [10]. In addition, as the wavelength increases, the depth of light penetration increases. Although this causes only a slight difference, it enables the induction of a more uniform photochemical reaction, thereby expanding the application. Consequently, photochemical reactions mediated by visible light have been extensively investigated in the biomedical field [11].

The wavelength of visible light used in photochemical reactions is determined by the absorption spectrum of the photoinitiator. Representative visible light-mediated photoinitiators include RF, eosin Y, camphorquinone, ruthenium bipyridine complex ([Ru(II)(bpy)_3_]^2+^), and lithium phenyl-2,4,6-trimethylbenzoylphosphinate (lithium aryl phosphinate (LAP)) (Table 1) [11]. Photoinitiators induce photochemical reactions by forming free radicals in the presence of light, and ensuring the cytocompatibility of such reactions caused by photoinitiators is a vital challenge in biomedical applications. LAP, RF, and eosin Y demonstrated cell viabilities >90% after combining the biopolymer precursor solution and cells, forming a hydrogel by visible light-induced crosslinking [11]. A direct comparison of the cytocompatibility of photoinitiators is challenging because cell viability is influenced by biopolymer materials and cell types. Several visible light-mediated photoinitiators suitable for biomedical applications are listed in Table 1.

LAP is a derivative of monoacylphosphine oxide (MAPO), which is modified to compensate for the disadvantages of MAPO-based photoinitiators, such as low water solubility [12,21]. LAP is frequently employed as a visible-light-mediated photoinitiator because it absorbs a wavelength of 405 nm. However, its absorption efficiency for visible light is significantly lower than that at 365 nm [22]. Because LAP contains lithium ions, it may adversely affect cells susceptible to lithium toxicity; however, the cytotoxicity of LAP can be reduced by adjusting the initiator concentration and light exposure parameters [23,24].

Camphorquinone, which is an aliphatic α-ketone, is a common photoinitiator. However, its efficiency as a sole agent is insufficient for applications; therefore, it is used in conjunction with a tertiary amine co-initiator that can provide electrons. Camphorquinone exhibits poor water solubility, which is a limiting factor because the majority of biopolymer crosslinking occurs in aqueous environments [25].

Ruthenium bipyridine complexes exhibit strong absorption properties at 450 nm and have been investigated as photoredox catalysts [26]. Although concerns exist regarding the potential toxicity of the ruthenium complex [27], it has been employed to form three-dimensional (3D) cell culture scaffolds with a high cell viability [17,18].

Eosin is a xanthene dye photosensitizer that can be used alone or as a co-initiator with triethanolamine [28]. Several properties distinguish eosin Y from other photoinitiators that can be mediated by visible light. First, eosin Y absorbs green light with a maximum absorption wavelength of 515 nm. Therefore, compared to the initiator absorbing at a wavelength of 400 nm, it exhibits an increased penetration. In addition, among the visible light-mediated photoinitiators listed in Table 1, water solubility of eosin Y is the highest, resulting in the fewest restrictions on application concentrations [28].

RF is a natural compound existing in nature and is a form of vitamin B. Consequently, it is beneficial to cells and exhibits good biocompatibility. Therefore, it has been employed as a photoinitiator in biomedical applications [29]. The cell viabilities listed in Table 1 are high, owing to the application of appropriate concentrations of photoinitiators. Based on an analysis of the cytotoxicity of photoinitiators, the cytocompatibility of RF is higher than that of other photoinitiators at the same concentration [11].

**Figure 1:**
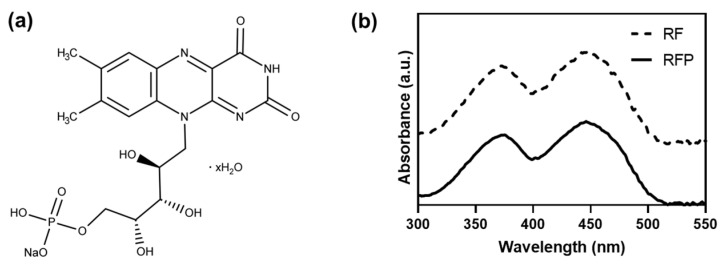
(**a**) Chemical structure of riboflavin phosphate. (**b**) Absorbance spectra of riboflavin (RF) and riboflavin phosphate (RFP) aqueous solution.

The water solubility of RF renders it suitable for application as a photoinitiator. Significantly, increasing the solubility of RF can increase its utility. RFP is frequently used as an alternative to RF to overcome its low solubility. RFP is a phosphate sodium salt of RF, which is also known as flavin mononucleotide, and the presence of a phosphate group significantly increases the water solubility to approximately 30 mg mL^−1^ (Figure 1a) [30]. Although RF and RFP have different water solubilities, they exhibit similar photochemical properties, such as the excitation wavelength, emission wavelength, and intensity; these characteristics render them useful as photoinitiators (Figure 1b) [31]. Both RF and RFP solutions are greenish yellow in color, and they have the same photochemical properties by sharing the flavin core structure. Additionally, the flavin group is based on isoalloxazine ring, a heterocyclic three-ring structure [32]. RF and RF carry out electron transfer reactions with proton transfer via the redox-active isoalloxazine system [33].

The advantage of RFP is that it is a photoinitiator that has already been approved by FDA and is used in medical facilities [34,35]. In ophthalmic applications, a combination of RFP and UV irradiation is employed to treat corneal keratoconus by inducing collagen crosslinking. Although the UV spectral range was FDA-approved, blue light can be used as a light source because RFP absorbs both the UV radiations and light with a wavelength of 444 nm.

### 2.2. Mechanism of Riboflavin-Mediated Photochemistry

The photochemical processes of RF and RFP originate in the flavin core. Considering the UV to the visible light spectral range, RF exhibits maximum absorbance at four wavelengths: 223, 267, 373, and 444 nm, in an aqueous solution with a pH of 7 [36,37]. RF exhibits fluorescence and emits light with a wavelength of 520 nm. At neutral pH, it exhibits robust fluorescence emissions and maintains these properties between pH values of 4 and 10 [38]. Protonation occurs below pH 4, and deprotonation occurs above pH 10; the photochemical properties are maintained between pH 4 and 10.

**Figure 2:**
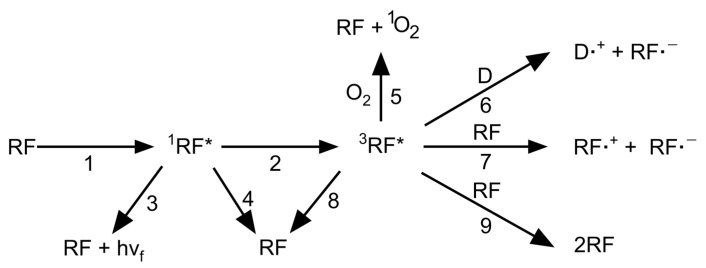
Schematic of the mechanistic pathways for the formation of intermediates by RF. Adapted with permission from [39].

RF functions as a visible light-mediated photoinitiator by absorbing light to create a reactive intermediate (Figure 2). The absorption of the light with a wavelength of 444 nm leads to photolysis, yielding a photo-degraded product, which then induces the formation of a reactive species [39]. In the presence of light, RF absorbs the as-formed product and is excited into a high-energy singlet state (^1^RF*). Subsequently, an excited triplet state (^3^RF*) is achieved through nonradiative intersystem crossing. Several singlet-state RFs do not reach the triplet state; their energy is released as heat or fluorescence (radiationless transitions). A portion of the produced ^3^RF* relaxes to the ground state. Crosslinking is induced by the energy transfer of RF in the triplet state, wherein the energy transfer can occur between neighboring molecules. When oxygen serves as an energy transfer mediator, the formation of singlet oxygen (^1^O_2_) triggers an oxidation reaction by transferring energy to an electron donor, such as a specific functional group of the biopolymer. Consequently, ^3^RF* is converted into a semi-reduced radical (**˙**PS^–^), and the donor forms a semi-oxidized radical (**˙**D^+^). RF may serve as a donor. Thiol, tyramine, and furfuryl functional groups act as donors and cause crosslinking.

The mechanism by which RF forms an active intermediate after light exposure can be extended to other typical photosensitizers. A triplet-state photosensitizer transfers energy through various routes to induce chemical structural changes. Crosslinking can be induced using a biopolymer capable of forming an interconnection or a biopolymer into which a functional group is introduced, which can be applied in biomedical applications.

## 3. Riboflavin-Induced Photochemistry

### 3.1. Oxidation

**Figure 3:**
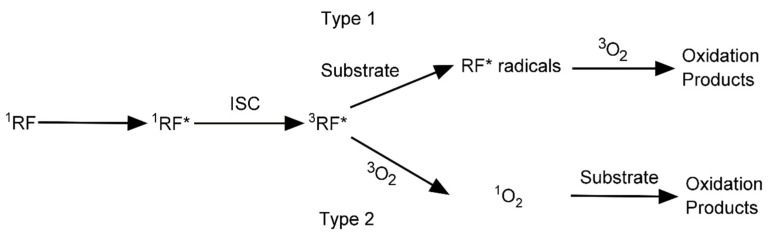
Schematic of the mechanism pathways for oxidation by RF. Adapted from [40].

The RF-mediated oxidation process transforms the substrate into an oxidation product that uses oxygen as a mediator (Figure 3) [40]. An oxidation product is formed when the energy of RF, excited to the triplet state, is transferred to oxygen. Bonds are then formed between the biopolymers by oxidation, and crosslinking is induced. Depending on the object to which the RF energy is transferred, two types of pathways exist for oxidation product formation. In Type 1, the RF transfers the excited energy to the substrate to form RF-free radicals. Subsequently, the free radicals interact with ground-state oxygen molecules to generate oxidation products. In Type 2, the energy of the excited RF is transferred to the oxygen molecules to form singlet molecular oxygen, which then reacts with the substrate to produce an oxidation product. Type 1 is the preferred pathway when the oxygen concentration is low, whereas the singlet molecular oxygen generated in the Type 2 pathway is more reactive than the RF radicals [41].

RF-induced oxidation reactions are involved in various biological redox processes. Reactive functional groups on biomolecules react with one another to form crosslinks when reactive oxygen species (ROS), **˙**OH, **˙**O_2_^2−^, and **˙**O_2_^−^, are produced from Type 2 reactions [37]. Protein crosslinking can be achieved by inducing the bonding of amino acids that constitute proteins. Histidine converts the imidazole moiety to electrophilic imidazolone by inducing ROS and reacts with nucleophilic amino acids, such as lysine, to form covalent crosslinks [42,43]. In addition to histidine–lysine, crosslink formation between the amino acids to form tyrosine–tyrosine, tyrosine–tryptophan, and tyrosine–lysine is possible under oxidative conditions [44].

RF-induced oxidation is used to crosslink proteins rich in amino acids, such as histidine, lysine, tyrosine, and tryptophan. Certain amino acids, including cysteine, methionine, tyrosine, tryptophan, and arginine, undergo oxidation in the presence of RF. In addition, several biomolecules, such as ascorbate and nicotinamide adenine dinucleotide hydrogen, in tissue can serve as electron donors to the PS triplet excited states without oxygen mediation [39]. Collagen crosslinking is a widely employed crosslinking method for RF and RFP. It is specifically used in medicine as a healing technique for keratoconus corneas. The collagen crosslinking of the corneal stromal layer is induced by light irradiation after RFP is applied to the layer [42,45]. In clinical treatments, UV light with a wavelength of 365 nm, which has a lower transmittance than visible light in the blue wavelength region, is employed to induce crosslinking at depths <200–300 μm. UV irradiation with a weak intensity of 3 mW cm^−2^ is applied for a period of approximately 30 min. Kang et al. demonstrated that RFP-induced crosslinking increased the Young’s modulus and tensile strength of skin tissues by approximately three times by applying the same procedure to skin collagen as that for corneal collagen [46]. Blue light is safer than UV light when applied to the skin; therefore, visible light with a wavelength of 450 nm was employed. In addition, the irradiation time was decreased to 5 min using a high intensity of 100 mW cm^−2^.

Kim et al. performed the RFP-induced crosslinking of keratin proteins [47]. The thiol group of the cysteine residue, which is abundant in keratin, participated in crosslinking using photoexcited RFP, and the crosslinking efficiency was increased using citric acid as a crosslinker. The resulting product could be used as an environmentally friendly personal care product, demonstrating significant effectiveness in the setting and strengthening of the hair by inducing crosslinking.

Because RF-mediated protein crosslinking, which is achieved by inducing oxidation, is typically performed by binding amino acid residues, it has the advantages of crosslinking, which can be performed without protein modification. However, the crosslinking density differs owing to the different amino acid compositions of various proteins. Additionally, its application to biopolymers is challenging, except for proteins such as polysaccharides. Another drawback is that the degree of crosslinking cannot be precisely controlled. However, if the appropriate conditions are determined and applied to obtain the required degree of crosslinking, it can be employed for crosslinking proteins in various tissues constituting the human body.

### 3.2. Thiol-Ene Reaction

The thiol-ene reaction, which is frequently referred to as photo-click chemistry, is a versatile reaction owing to the high selectivity between thiol and vinyl groups. The thiol group loses a proton after the initiator is activated, forming a thiyl radical [48]. This radical then reacts with the vinyl bond, resulting in the formation of a thioester bond.

The type of alkene used in the thiol-ene reaction affects the reaction rate and properties, such as alkene conversion. Norbornenes are common alkenes used in thiol-ene reactions. Norbornene forms a thioester bond with a thiol group through a low-energy reaction, resulting in a rapid reaction with a high conversion rate [48]. However, the light stability is low, and the selectivity for progressing the reaction by irradiating light at a particular time decreases in terms of the light-responsive reaction.

**Figure 4:**
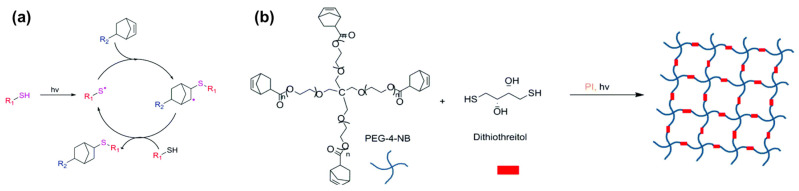
(**a**) Schematic of the thiol-ene reaction mechanism. (**b**) Schematic of the hydrogelation using 4-arm PEG-norbornene and DTT. Reprinted with permission from [49].

Thiol-ene reactions are frequently employed after modifying the target biomaterials with thiol or alkene groups because they can promote crosslinking when paired with thiols and alkenes. Therefore, synthetic polymers, which are more straightforward to modify than natural biopolymers, are frequently used. For example, the synthetic polymer, poly(ethylene glycol) (PEG), has been crosslinked via the thiol-ene reaction. Stenzel’s group created a crosslinked hydrogel by reacting 4-arm PEG-norbornene with dithiothreitol (DTT) [49,50]. When RFP is exposed to light, the excited RFP removes hydrogen from the thiol group of DTT to form a thiyl radical. This radical then reacts with the alkene of norbornene to form a thioether linkage (Figure 4).

Methacrylate is another type of alkene used in thiol-ene reactions. It is more dependent on the light energy because it requires more energy for thiol-ene reactions than norbornene. Therefore, the reaction can proceed selectively when blue light is irradiated using RFP as the photoinitiator. Previous studies have demonstrated that the reaction progress varies depending on the RFP concentration [51]. RFP concentrations between 0.001% and 0.1% were investigated, and the highest acceleration of gelation was achieved using 0.01% RFP. An RFP concentration of 0.001% was insufficient to initiate treatment. At 0.1%, the dark yellow color of RFP prevented the penetration of blue light, leading to insufficient gelation. Therefore, the key to regulating the gelation rate is to use an appropriate amount of RFP.

Methacrylated biopolymers often have been crosslinked by photoinitiation of RF without a thiol group. Due to the lack of photoinitiation energy of RFP, methacrylated biopolymers typically require RFP as well as an extra co-initiator for vinyl crosslinking. Goto et al. crosslinked methacrylated gelatin (GelMA) using RF with triethanolamine as a co-initiator [52]. In terms of GelMA hydrogel properties, the combination of RF and visible light-induced crosslinking was comparable to that of Irgacure2959 and UV.

Hyaluronic acid (HA) is a crosslinked biopolymer created by the reaction of thiol groups with methacrylate [51,53]. HA is a biocompatible polymer that promotes cell migration and proliferation, and it reduces water evaporation owing to its attraction to water. HA interacts with CD44, which is involved in autoimmune diseases and cancer [54]. In addition, HA is occasionally used in combination with medications because of its therapeutic effects [53]. After introducing methacrylate and thiol groups to HA, the two HA components were mixed with RFP and exposed to blue light to produce an HA hydrogel via the thiol-ene reaction [51]. Because the thiol-ene reaction between the methacrylate and thiol groups is slower than that between the norbornene and thiol groups, a higher intensity or longer irradiation time is required. Abdul-Monem et al. utilized RFP to crosslink methacrylated HA without the thiolated HA part, and dimethylaminoethyl methacrylate was added as a co-initiator [55].

The thiol-ene reaction is a light-responsive crosslinking reaction that is more selective than the oxidation reaction; therefore, it is an appropriate method for inducing crosslinking between specific biomolecules. The oxidation reaction requires an irradiation time of several minutes to several tens of minutes, whereas the thiol-ene reaction can induce sufficient crosslinking within several tens of seconds to several minutes. Although the reactivity varies depending on the type of alkene, the reaction rate between the thiol and alkene groups can be significantly increased by the photoinitiation of RFP. In addition, it does not produce any reaction by-products and is not sensitive to O_2_, rendering it suitable for application in living organisms.

### 3.3. Tyramine and Tyrosine Groups

**Figure 5:**
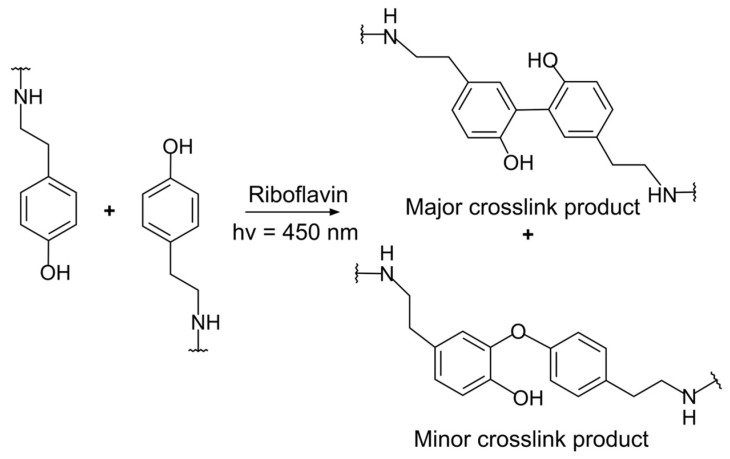
Schematic of the dityramine formation via the RF-mediated photoreaction from tyramine groups.

Tyramine or tyrosine groups create crosslinks by forming dityramine or dityrosine via excited RF (Figure 5). Tyrosyl radicals are created by the tyrosine group when RF is excited, and these radicals then form carbon–carbon or carbon–oxygen bonds to create dityrosine bonds [39]. Both tyramine and tyrosine possess a phenyl group, which acts as an electron donor and bond-forming site; therefore, the same mechanism can be applied. Tyrosine, which is an amino acid building block, is assumed to contribute to the oxidation mechanism that causes protein crosslinking. Tyramine is simple to conjugate through various chemical processes because it contains a primary amine group at its terminal. Therefore, tyramine can be used to form a pendant phenyl group in a biopolymer, and RF photosensitization initiates the crosslinking.

Kim et al. synthesized tyramine-modified alginate by conjugating tyramine to alginate using 1-ethyl-3-(3-dimethylaminopropyl) carbodiimide hydrochloride (EDC)/N-hydroxysulfosuccinimide (NHS) chemistry [56]. Dityramine was produced after 3 min of exposure to visible light at 440 nm and 2500 mW cm^−2^. It has been demonstrated that dual crosslinking can be employed to regulate the mechanical properties of alginate hydrogel because alginate possesses ionic crosslinking capabilities. Hong et al. synthesized tyramine-modified HA using EDC/NHS chemistry to prepare HA hydrogels [57,58]. RFP and sodium persulfate (SPS) were employed as the photoinitiator and co-initiator, respectively, to induce photocrosslinking, which was achieved by exposing the material to visible light at 2500 mW cm^−2^ and 440 nm for 30 s. The resulting mechanical strength of the hydrogel was comparable to that of the commercial filler, Restylane, and an in vitro cytocompatibility assay demonstrated that the dityrosine crosslinking caused by RFP was biocompatible.

Silk fibroin is a biocompatible and biodegradable biopolymer and is used as an optical element owing to its high transparency [59]. Applegate et al. demonstrated that photoexcited RF formed a dityrosine bond between the tyrosine residues of silk fibroin, and a high-resolution pattern of approximately 50 μm was fabricated via photolithography [60]. Piluso et al. manufactured a silk fibroin hydrogel that could be used in tissue engineering and cell delivery applications through dityrosine crosslinking [61]. Tyrosine residues, which constitute approximately 9% of the hydrophobic blocks in silk fibroin, were used without any additional chemical modifications. RF and SPS were used as the photoinitiator and co-initiator, respectively, to induce the reaction. Visible light in the 400–700 nm range was used as the light source for 10 min. The silk fibroin hydrogel exhibited a storage modulus > 10^3^ Pa and led to a cell viability of >80% even after one week of cell encapsulation in the hydrogel. Silk fibroin has the advantage of forming dityrosine crosslinks without additional chemical modifications.

The formation of tyrosyl radicals from the tyrosine group using excited RF is a type of amino-acid oxidation. RF-mediated oxidative crosslinking results from a random combination of oxidation products of amino acid residues contained in proteins. Although all amino acid residues cannot create links [62], a higher crosslinking efficiency can be achieved because tyrosine creates a dityrosine between tyrosine residues to form a specific link.

### 3.4. Furfuryl Group

**Figure 6:**
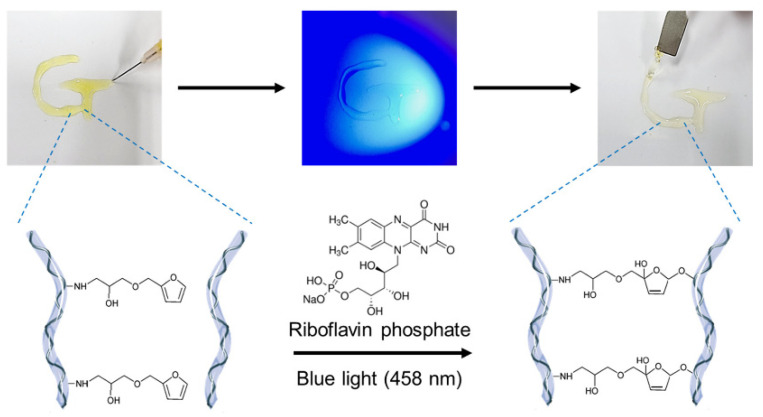
Photocrosslinking mechanism of the furfuryl group under blue light irradiation using RFP. Reprinted from [64].

The furfuryl group is a chemical structure comprising a furan ring and has a visible-light-reactive functional group. Furfuryl groups are crosslinked by forming furan endoperoxides through a photo-oxidation crosslinking mechanism in the presence of singlet oxygen (Figure 6) [63,64]. Because the furfuryl group is not a functional group found in natural biopolymers, the chemical modification is essential. Therefore, selective reactivity to light can be imparted by introducing a furfuryl group into the biopolymer.

Gelatin, after functionalization with a furfuryl group, is widely employed as a biopolymer. As a partially hydrolyzed collagen, gelatin is a preferred biopolymer in the biomedical industry because of its biocompatibility and biodegradability. Kong et al. created an injectable gelatin hydrogel by adding a furfuryl group to gelatin, which can be employed as a controlled drug delivery system [64]. Selective bonding was used to create the drug delivery system by combining the furfuryl and maleimide groups, representing a type of Diels–Alder click chemistry. The epidermal growth factor (EGF) was conjugated to a maleimide group and then linked to furfuryl-modified gelatin through a Diels–Alder reaction. Hydrogels were then created using the remaining furfuryl groups by crosslinking the gelatin. Chemically immobilized EGF in the gelatin matrix was sustainably released from the hydrogel. 

Joddar et al. demonstrated the applicability of furfuryl-modified gelatin as a tissue engineering scaffold [65,66]. The cell viability, network formation, and proliferation were excellent after the cells were enclosed in a 3D gelatin scaffold with 400 nm light irradiation [65]. In addition, a nanofiber scaffold was formed using furfuryl-modified gelatin by electrospinning and exhibited mechanical properties similar to those of the extracellular matrix found in cardiac tissues [66]. The utility of photocrosslinked gelatin electrospun fibers for tissue engineering was demonstrated by the adhesion and growth of cardiomyocytes and induced pluripotent stem cells.

Heo et al. introduced a furfuryl group into alginate to produce visible-light-curable alginate and employed it as a controlled release system [67,68]. RF-mediated photocrosslinked alginate hydrogel exhibited mechanical and release properties similar to those of alginate hydrogels physically crosslinked by calcium ions, demonstrating that it is appropriate for application in drug delivery systems [67]. In addition, alginate has been employed as a visible-light-curable anti-adhesion agent because it exhibits non-adhesive properties in tissues [68].

However, a limitation of the aforementioned approach is that the furfuryl group can only be used when it is introduced into a biopolymer for the RF-mediated photocrosslinking reaction. However, because it has been introduced into various biopolymers, as mentioned previously, the furfuryl group is a promising candidate for application in the biomedical industry. The storage stability of the thiol group participating in the thiol-ene reaction, which also requires functional group modification, is inferior to that of the furfuryl group. However, because the thiol-ene reaction is better in terms of photoreactivity, the RF-mediated mechanism can be selected based on the required purpose.

We summarized the RF-induced photochemistry with applied biomaterials and light irradiation conditions in Table 2.

## 4. Biomedical Applications

Significant efforts have been devoted to applying RF-mediated photocrosslinking in biomedicine. Considering the photoinitiators that can be employed in biomedical applications, RF is a promising candidate because it is a natural product as well as a vitamin. In this section, biomedical applications are divided into four categories: tissue crosslinking, drug delivery, dermal filler, and cellular scaffolds for tissue engineering (Table 3).

### 4.1. Tissue Crosslinking

The oldest and most widely employed method of RF-mediated photocrosslinking is tissue crosslinking [34,35]. The light source used for the excitation of RF is UV or blue light. Herein, the depth at which the light penetrates the tissue is small, such that its use is limited to the cornea, skin, and hair, when irradiated on the human body surface. Tissue crosslinking is derived from linkages between proteins, such as collagen or keratin, that comprise tissues via oxidation photochemistry.

The cornea is a frequently used tissue in clinical practice for RF-mediated crosslinking [34,35]. Clinically, corneal crosslinking, which involves exposing RFP to UVA light in the 365–370 nm range, is used to treat keratoconus. Keratoconus is a condition in which the corneal stromal collagen density weakens, changing the refractive index and impairing vision. The robustness of the corneal stroma can be increased using treatments. UV light is considered a suitable light source for targeted cornea photochemistry, and a low UV intensity with a long irradiation time is employed. Efforts to utilize safer light sources and shorter irradiation times are expected to continue.

Similar to the cornea, skin tissue is exposed to the environment, and collagen is a significant component of the dermis. Applying RFP-mediated photochemistry to skin tissue increases its elasticity and strength [46]. For practical applications, further studies are required to achieve the delivery of sufficient amounts of RFP to the dermal layer for photochemistry. In addition, further studies are required to ascertain the level of skin collagen strengthening that positively affects the skin. Although not yet used in clinics or esthetics, it is a promising application because it can be offered to more people than corneal strengthening after ensuring its safety through further studies.

Keratin protein crosslinking has been demonstrated to be effective in strengthening and setting hair [47]. Compared with permanent hair waving methods that use chemicals such as thioglycolic acid, it has limitations in maintaining hair curls. However, this safe one-step crosslinking process uses RFP and other environmentally friendly chemicals. The green chemistry method for hair treatment will gain attention with an improvement in the hair-setting effect.

Tissue crosslinking uses an oxidation mechanism without further biopolymer modification. Most tissue crosslinking is already widely used in practical applications or will soon be employed. Because light irradiation must be performed over an extended period with a low crosslinking rate, the oxidation method can be further improved to decrease the irradiation time and increase the crosslinking density.

### 4.2. Drug Delivery

An advantage of RF-mediated photocrosslinked hydrogels is the in situ crosslinking by light irradiation. Most precursor solutions can be injected into tissues, such as skin and cornea, as a low-viscosity liquid phase before gelation begins. After injecting the drug-containing precursor solution through a syringe, light irradiation creates a biopolymer hydrogel containing the drug at the desired location. The drug can be released in a small area over an extended period from the in situ crosslinked hydrogels.

Heo et al. observed the insulin-like growth factor-1 (IGF-1) release behavior after loading it into an RF-mediated crosslinked alginate hydrogel [67]. Cell viability was observed to be unaffected by the fabrication process and photocrosslinked alginate hydrogel, and the sustained release of IGF-1 promoted cell growth. Son et al. employed alginate and HA as biopolymer matrices and used them as anti-adhesion agents [68,70]. An anti-adhesion effect was produced by creating a physically protective biopolymer biofilm at the surgical site. In addition, an anti-inflammatory function was achieved through ibuprofen delivery.

A controlled release of materials is possible owing to the properties of the functional groups participating in RFP-mediated crosslinking. Kong et al. reported that maleimide and Diels–Alder reactions were performed in conjunction with the furfuryl group introduction for crosslinking, resulting in a covalent bond between the maleimide-modified EGF and the gelatin matrix [64]. A method for long-term release without burst release was proposed because the drug release rate was not dependent on drug diffusion from inside the hydrogel to the outside, but instead released upon matrix degradation.

Most studies employed an auxiliary form of mixing a drug into a biopolymer precursor solution for applications other than drug delivery [71]. In future, it is expected that various drugs can be introduced to add additional functions to photocrosslinked hydrogels. In addition, extensive studies into controlled-release systems will be conducted through various applications of functional groups introduced for RF-mediated crosslinking.

### 4.3. Dermal Filler

HA is a biopolymer used as an injectable dermal filler owing to its unique viscoelastic properties. A current limitation of HA fillers is the prolonged post-implantation period, which can be overcome by crosslinking. However, when the crosslinking density is increased to the required level, it is challenging to deliver crosslinked HA through a syringe. Therefore, RF-mediated photochemistry can be used as an alternative to crosslinking after the HA solution is injected into the target site.

Kim et al. demonstrated that the degradation rate of the crosslinked HA hydrogel was significantly decreased by crosslinking HA in a biocompatible manner through an RFP-mediated thiol-ene reaction [53]. Blue light penetrated the porcine skin and reached the HA hydrogel precursor solution, and an HA hydrogel was successfully formed. Therefore, the feasibility of HA injection into the dermis layer for crosslinking was demonstrated. In addition, it was possible to simultaneously achieve the functions of the filler and drug delivery by incorporating EGF into the HA hydrogel.

Hong et al. introduced tyramine into HA to form an HA hydrogel that could be used as a soft tissue filler through RFP-mediated photocrosslinking [58]. An excellent compression recovery of >80% was achieved, and the compressive strength was approximately 130 kPa. In addition, prolonged exposure to blue light improves physiological stability while reducing biodegradation via enzymatic degradation using hyaluronidase.

Although the potential of injectable fillers with RF-mediated in vivo photochemistry has not yet been demonstrated, in vitro cell experiments have exhibited excellent cytocompatibility. Commercialization will be feasible when the mechanical properties to facilitate the required filler effect are optimized by in vivo testing.

### 4.4. Cellular Scaffolds for Tissue Engineering

Crosslinked biopolymers used in tissue engineering frequently contain cells in a hydrogel matrix. Therefore, photochemical crosslinking directly exposes cells to photoinitiators and light. The most common method involves the use of a photoinitiator that reacts with the UV irradiation. However, when RF and blue light are combined, adverse effects on the cells can be reduced.

Piluso et al. evaluated the cytocompatibility of RF-mediated photochemistry for fabricating silk hydrogels using articular cartilage-derived progenitor cells, mesenchymal stem cells, and dental pulp-derived stem cells [61]. All cells were confirmed to possess a viability of ≥80%, and it was demonstrated that this viability could be used for tissue engineering and cell delivery applications.

Anilkumar et al. demonstrated the potential of furfuryl-modified gelatin as a bioink for engineering complex tissues [65]. A gel structure was rapidly formed with visible-light irradiation for approximately 2.5 min. A hetero-cellular cluster with a bilayer structure was created by the coculture of cardiac fibroblasts and cardiomyocytes, demonstrating the potential to create a functional cardiac patch.

Cellular scaffold applications require a short gelation period because gelation must be performed with cells, so highly reactive RF-mediated photochemistry, such as thiol-ene, tyramine, and furfuryl groups, are suitable. Therefore, synthetic polymers, such as PEG, are employed more frequently than natural biopolymers because the modification of the polymer matrix is necessary [49,50]. In future, an increase in the use of biopolymers is predicted owing to their environmental benefits and biocompatibility. Particularly, RF-mediated photochemistry possesses qualities that make it a suitable choice for use as a cell-laden bioink for bioprinting in tissue engineering. The best option for photocrosslinking in the bioprinting process can be RF because of its quick crosslinking with short irradiation time and few adverse effects from the photoinitiator.

## 5. Perspectives

### 5.1. Challenges in Riboflavin-Mediated Photochemistry for Clinical Applications

RF-mediated photochemistry shows promise for clinical applications; however, several issues must be resolved. First, the transmittance of light to the tissue must be improved. Despite significant progress in optics, the fundamental properties of light prevent transmittance beyond a few millimeters. Light transmission is unhindered in ex vivo applications; however, when used in clinical settings, it must pass through the skin, and short-wavelength light, such as UV and blue light, shows poor skin penetration. Light penetration depth is determined by the degree of absorption and scattering of light in the cells or extracellular matrix constituting the tissue. The wavelength range of light that can effectively penetrate the biological tissue or skin is termed as the ‘photodynamic therapy (PDT) window’, which is typically between 650 and 950 nm [8]. Light in the PDT window minimizes absorption or scattering by tissues or biomolecules, thereby enabling deep penetration. However, achieving a centimeter-scale penetration depth is challenging. The limitations of in vivo applications will remain until a novel clinical technique is developed that can deliver light to the interior of the body. However, it is anticipated to be applicable in near future because light transmission is not limited in integumentary tissues, such as the cornea and skin, which are exposed on the outer surface of the body.

In RF-based photochemistry, RF is not a photoinitiator that undergoes homolytic photodissociation to generate radicals [39]. The energy of RF molecules excited by light irradiation can be transferred to increase the efficiency of the photochemical reaction when a co-initiator that can supply electrons is used in tandem. In previous studies, photochemistry increased efficiency using triethanolamine or *L*-arginine containing an electron donor group, amine, or amino group as a co-initiator [72,73]. The type of co-initiator that can be used and the appropriate concentration level may be restricted, depending on the biomedical application. In other words, it is crucial to investigate and employ RF and co-initiator optimization based on the requirements of each application.

### 5.2. Future of Riboflavin-Mediated Photochemistry

Photocrosslinking has an immediate on/off response with a significant response compared to that toward other triggers, such as heat or pH. In contrast to ultrasound and magnetic fields, photocrosslinking can also be used locally. In other words, it has the advantage of enabling fine control through the adjustment of the irradiation intensity, wavelength, and time of the light source in a local area. Although various photoinitiators can be used to induce photochemistry, a promising advantage of RF is that it is a natural compound. It is challenging to determine that whether the photoinitiation efficiency of RF is superior to that of other photoinitiators. However, it is among the most advanced candidates in terms of biocompatibility. The optimization of certain factors, including the functional group type, RF concentration, light source intensity, and light delivery techniques, are necessary for facilitating its widespread application in the biomedical and clinical fields. In future, the application of RF-based photochemistry will significantly increase as the importance of environmentally friendly products and procedures increases.

## Figures and Tables

**Table 1:** Types of visible light-mediated photoinitiators. Adapted with permission from [11].

Photoinitiator	Chemical Structure	Solubility in Water	Absorption Wavelength (nm)	Cell Viability (1 d)	Refs.
LAP	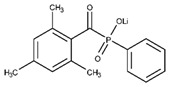	<30 mg mL^−1^	405	>90%	[12,13]
RF	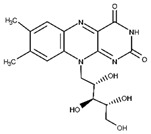	<0.5 mg mL^−1^	444	>90%	[14,15]
Camphorquinone	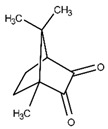	Slightly soluble	450	50–90%	[14,16]
[Ru(II)(bpy)_3_]^2+^ *	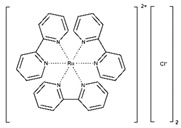	Slightly soluble	452	85–90%	[17,18]
Eosin Y	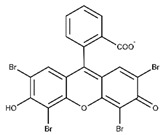	<50 mg mL^−1^	515	>96%	[19,20]

* Tris(2,2′-bipyridyl)dichlororuthenium(II) hexahydrate.

**Table 2:** Types of RF-induced photochemistry and crosslinked biomaterials.

Photochemistry	Biopolymer	Photoinitiator	Light Source Wavelength (nm)	Light Intensity (mW cm^−2^)	Irradiation Time	Refs.
Oxidation	Collagen (Cornea)	RF	405	3	30 min	[42,45]
	Collagen (Skin)	RFP	450	100	5 min	[46]
	Keratin (Hair)	RFP	450	3	5 min	[47]
Thiol-ene reaction	HA	RFP	450	100	40 s	[51,53]
Tyramine group	Alginate	RF	440	2500	3 min	[56]
	HA	RFP	440	2500	30 s	[57,58]
	HA	RF	365	10	20 min	[69]
	Silk fibroin	RF	450	18.7	20 min	[60]
	Silk fibroin	RF	400–700	-	10 min	[61]
Furfuryl group	Gelatin	RFP	458	100	10 min	[64]
	Gelatin	RF	400	-	2.5 min	[65,66]
	Alginate	RF	445	200	10 min	[67,68]
	HA	RF	-	-	10 min	[70]

**Table 3:** Biomedical applications of RF-induced photochemistry.

Application	Biopolymer	Photoinitiator	Photochemistry	Refs.
Tissue crosslinking	Collagen (Cornea)	RF and RFP	Oxidation	[34,35]
	Collagen (Skin)	RFP	Oxidation	[46]
	Keratin (Hair)	RFP	Oxidation	[47]
Drug delivery	Alginate	RF	Furfuryl group	[67,68]
	HA	RF	Furfuryl group	[70]
	Gelatin	RFP	Furfuryl group	[64]
Dermal filler	HA	RFP	Thiol-ene reaction	[53]
	HA	RFP	Tyramine group	[58]
Cellular scaffolds for tissue engineering	Silk fibroin	RF	Tyramine group	[61]
	Gelatin	RF	Furfuryl group	[65]

## Data Availability

Not applicable.

## References

[1] Arif Z.U., Khalid M.Y., Sheikh M.F., Zolfagharian A., Bodaghi M. (2022). Biopolymeric sustainable materials and their emerging applications. J. Environ. Chem. Eng..

[2] Qi X., Tong X., Pan W., Zeng Q., You S., Shen J. (2021). Recent advances in polysaccharide-based adsorbents for wastewater treatment. J. Clean. Prod..

[3] Sun J., Shen J., Chen S., Cooper M.A., Fu H., Wu D., Yang Z. (2018). Nanofiller Reinforced Biodegradable PLA/PHA Composites: Current Status and Future Trends. Polymers.

[4] Alaswad S.O., Mahmoud A.S., Arunachalam P. (2022). Recent Advances in Biodegradable Polymers and Their Biological Applications: A Brief Review. Polymers.

[5] Mitura S., Sionkowska A., Jaiswal A. (2020). Biopolymers for hydrogels in cosmetics: Review. J. Mater. Sci. Mater. Med..

[6] Reddy N., Reddy R., Jiang Q. (2015). Crosslinking biopolymers for biomedical applications. Trends Biotechnol..

[7] Badeau B.A., Comerford M.P., Arakawa C.K., Shadish J.A., DeForest C.A. (2018). Engineered modular biomaterial logic gates for environmentally triggered therapeutic delivery. Nat. Chem..

[8] Rapp T.L., DeForest C.A. (2020). Visible Light-Responsive Dynamic Biomaterials: Going Deeper and Triggering More. Adv. Healthc. Mater..

[9] Burdick J.A., Murphy W.L. (2012). Moving from static to dynamic complexity in hydrogel design. Nat. Commun..

[10] Khoshakhlagh P., Bowser D.A., Brown J.Q., Moore M.J. (2019). Comparison of visible and UVA phototoxicity in neural culture systems micropatterned with digital projection photolithography. J. Biomed. Mater. Res. Part A.

[11] Zheng Z., Eglin D., Alini M., Richards G.R., Qin L., Lai Y. (2021). Visible Light-Induced 3D Bioprinting Technologies and Corresponding Bioink Materials for Tissue Engineering: A Review. Engineering.

[12] Fairbanks B.D., Schwartz M.P., Bowman C.N., Anseth K.S. (2009). Photoinitiated polymerization of PEG-diacrylate with lithium phenyl-2,4,6-trimethylbenzoylphosphinate: Polymerization rate and cytocompatibility. Biomaterials.

[13] Wang Z., Jin X., Dai R., Holzman J.F., Kim K. (2016). An ultrafast hydrogel photocrosslinking method for direct laser bioprinting. RSC Adv..

[14] Hu J., Hou Y., Park H., Choi B., Hou S., Chung A., Lee M. (2012). Visible light crosslinkable chitosan hydrogels for tissue engineering. Acta Biomater..

[15] Ibusuki S., Halbesma G.J., Randolph M.A., Redmond R.W., Kochevar I.E., Gill T.J. (2007). Photochemically cross-linked collagen gels as three-dimensional scaffolds for tissue engineering. Tissue Eng..

[16] Kamoun E.A., El-Betany A., Menzel H., Chen X. (2018). Influence of photoinitiator concentration and irradiation time on the crosslinking performance of visible-light activated pullulan-HEMA hydrogels. Int. J. Biol. Macromol..

[17] Al-Abboodi A., Zhang S., Al-Saady M., Ong J.W., Chan P.P.Y., Fu J. (2019). Printing in situ tissue sealant with visible-light-crosslinked porous hydrogel. Biomed. Mater..

[18] Bertlein S., Brown G., Lim K.S., Jungst T., Boeck T., Blunk T., Tessmar J., Hooper G.J., Woodfield T.B.F., Groll J. (2017). Thiol–Ene Clickable Gelatin: A Platform Bioink for Multiple 3D Biofabrication Technologies. Adv. Mater..

[19] Fu A., Gwon K., Kim M., Tae G., Kornfield J.A. (2015). Visible-Light-Initiated Thiol–Acrylate Photopolymerization of Heparin-Based Hydrogels. Biomacromolecules.

[20] Petta D., Grijpma D.W., Alini M., Eglin D., D’Este M. (2018). Three-Dimensional Printing of a Tyramine Hyaluronan Derivative with Double Gelation Mechanism for Independent Tuning of Shear Thinning and Postprinting Curing. ACS Biomater. Sci. Eng..

[21] Majima T., Schnabel W., Weber W. (1991). Phenyl-2,4,6-trimethylbenzoylphosphinates as water-soluble photoinitiators. Generation and reactivity of O=P(C_6_H_5_)(O^−^) radical anions. Die Makromol. Chem..

[22] Ki C.S., Shih H., Lin C.-C. (2013). Facile preparation of photodegradable hydrogels by photopolymerization. Polymer.

[23] Nguyen A.K., Goering P.L., Elespuru R.K., Sarkar Das S., Narayan R.J. (2020). The Photoinitiator Lithium Phenyl (2,4,6-Trimethylbenzoyl) Phosphinate with Exposure to 405 nm Light Is Cytotoxic to Mammalian Cells but Not Mutagenic in Bacterial Reverse Mutation Assays. Polymers.

[24] Efrati S., Averbukh M., Berman S., Feldman L., Dishy V., Kachko L., Weissgarten J., Golik A., Averbukh Z. (2004). N-Acetylcysteine ameliorates lithium-induced renal failure in rats. Nephrol. Dial. Transplant..

[25] Jakubiak J., Allonas X., Fouassier J.P., Sionkowska A., Andrzejewska E., Linden L.Å., Rabek J.F. (2003). Camphorquinone–amines photoinitating systems for the initiation of free radical polymerization. Polymer.

[26] Nicewicz D.A., MacMillan D.W.C. (2008). Merging Photoredox Catalysis with Organocatalysis: The Direct Asymmetric Alkylation of Aldehydes. Science.

[27] Carnizello A.P., Alves J.M., Pereira D.E., Campos J.C.L., Barbosa M.I.F., Batista A.A., Tavares D.C. (2019). Study of the cytotoxic and genotoxic potential of the carbonyl ruthenium(II) compound, ct-[RuCl(CO)(dppb)(bipy)]PF6 [dppb = 1,4-bis(diphenylphosphino)butane and bipy = 2,2′-bipyridine], by in vitro and in vivo assays. J. Appl. Toxicol..

[28] Shih H., Lin C.-C. (2013). Visible-Light-Mediated Thiol-Ene Hydrogelation Using Eosin-Y as the Only Photoinitiator. Macromol. Rapid Commun..

[29] Cheung I.M.Y., McGhee C.N.J., Sherwin T. (2014). Beneficial effect of the antioxidant riboflavin on gene expression of extracellular matrix elements, antioxidants and oxidases in keratoconic stromal cells. Clin. Exp. Optom..

[30] Bampidis V., Azimonti G., Bastos M.d.L., Christensen H., Dusemund B., Kouba M., Kos Durjava M., López-Alonso M., Puente S.L., EFSA Panel on Additives and Products or Substances used in Animal Feed (FEEDAP) (2018). Safety and efficacy of vitamin B2 (riboflavin 5′-phosphate ester monosodium salt) for all animal species when used in water for drinking. EFSA J..

[31] Heelis P.F. (1982). The photophysical and photochemical properties of flavins (isoalloxazines). Chem. Soc. Rev..

[32] Kotaki A., Yagi K. (1970). Fluorescence Properties of Flavins in Various Solvents. J. Biochem..

[33] Aleksandrov A. (2019). A Molecular Mechanics Model for Flavins. J. Comput. Chem..

[34] Wollensak G., Spoerl E., Seiler T. (2003). Riboflavin/ultraviolet-a–Induced collagen crosslinking for the treatment of keratoconus. Am. J. Ophthalmol..

[35] Raiskup F., Spoerl E. (2013). Corneal Crosslinking with Riboflavin and Ultraviolet A. I. Principles. Ocul. Surf..

[36] Sheraz M.A., Kazi S.H., Ahmed S., Anwar Z., Ahmad I. (2014). Photo, thermal and chemical degradation of riboflavin. Beilstein J. Org. Chem..

[37] Beztsinna N., Solé M., Taib N., Bestel I. (2016). Bioengineered riboflavin in nanotechnology. Biomaterials.

[38] Drössler P., Holzer W., Penzkofer A., Hegemann P. (2002). pH dependence of the absorption and emission behaviour of riboflavin in aqueous solution. Chem. Phys..

[39] Redmond R.W., Kochevar I.E. (2019). Medical Applications of Rose Bengal- and Riboflavin-Photosensitized Protein Crosslinking. Photochem. Photobiol..

[40] Gabriela I., Iulia M., Mohammed A.A.K. (2019). Application of Riboflavin Photochemical Properties in Hydrogel Synthesis. Biophysical Chemistry.

[41] Choe E., Huang R., Min D.B. (2005). Chemical Reactions and Stability of Riboflavin in Foods. J. Food Sci..

[42] McCall A.S., Kraft S., Edelhauser H.F., Kidder G.W., Lundquist R.R., Bradshaw H.E., Dedeic Z., Dionne M.J.C., Clement E.M., Conrad G.W. (2010). Mechanisms of Corneal Tissue Cross-linking in Response to Treatment with Topical Riboflavin and Long-Wavelength Ultraviolet Radiation (UVA). Investig. Ophthalmol. Vis. Sci..

[43] Shen H.-R., Spikes J.D., Kopečeková P., Kopeček J. (1996). Photodynamic crosslinking of proteins. I. Model studies using histidine- and lysine-containing N-(2-hydroxypropyl) methacrylamide copolymers. J. Photochem. Photobiol. B Biol..

[44] Mariotti M., Leinisch F., Leeming D.J., Svensson B., Davies M.J., Hägglund P. (2018). Mass-Spectrometry-Based Identification of Cross-Links in Proteins Exposed to Photo-Oxidation and Peroxyl Radicals Using 18O Labeling and Optimized Tandem Mass Spectrometry Fragmentation. J. Proteome Res..

[45] Hayes S., Kamma-Lorger C.S., Boote C., Young R.D., Quantock A.J., Rost A., Khatib Y., Harris J., Yagi N., Terrill N. (2013). The Effect of Riboflavin/UVA Collagen Cross-linking Therapy on the Structure and Hydrodynamic Behaviour of the Ungulate and Rabbit Corneal Stroma. PLoS ONE.

[46] Kang Y., Kim J.H., Kim S.Y., Koh W.-G., Lee H.J. (2021). Blue Light-Activated Riboflavin Phosphate Promotes Collagen Crosslinking to Modify the Properties of Connective Tissues. Materials.

[47] Kim S.Y., Kim J.H., Kang Y., Yoo J.W., Choi J., Lee H.J. (2022). Green chemistry method for hair strengthening and setting using visible light-mediated protein crosslinking. J. Clean. Prod..

[48] Northrop B.H., Coffey R.N. (2012). Thiol–Ene Click Chemistry: Computational and Kinetic Analysis of the Influence of Alkene Functionality. J. Am. Chem. Soc..

[49] Batchelor R.R., Kwandou G., Spicer P.T., Stenzel M.H. (2017). (−)-Riboflavin (vitamin B2) and flavin mononucleotide as visible light photo initiators in the thiol–ene polymerisation of PEG-based hydrogels. Polym. Chem..

[50] Monfared M., Nothling M.D., Mawad D., Stenzel M.H. (2021). Effect of cell culture media on photopolymerizations. Biomacromolecules.

[51] Lee H.J., Fernandes-Cunha G.M., Myung D. (2018). In situ-forming hyaluronic acid hydrogel through visible light-induced thiol-ene reaction. React. Funct. Polym..

[52] Goto R., Nishida E., Kobayashi S., Aino M., Ohno T., Iwamura Y., Kikuchi T., Hayashi J.-i., Yamamoto G., Asakura M. (2021). Gelatin Methacryloyl–Riboflavin (GelMA–RF) Hydrogels for Bone Regeneration. Int. J. Mol. Sci..

[53] Kim H., Koh W.-G., Lee H.J. (2021). Effects of basic fibroblast growth factor combined with an injectable in situ crosslinked hyaluronic acid hydrogel for a dermal filler. React. Funct. Polym..

[54] Puré E., Cuff C.A. (2001). A crucial role for CD44 in inflammation. Trends Mol. Med..

[55] Abdul-Monem M.M., Kamoun E.A., Ahmed D.M., El-Fakharany E.M., Al-Abbassy F.H., Aly H.M. (2021). Light-cured hyaluronic acid composite hydrogels using riboflavin as a photoinitiator for bone regeneration applications. J. Taibah Univ. Med. Sci..

[56] Kim E., Kim M.H., Song J.H., Kang C., Park W.H. (2020). Dual crosslinked alginate hydrogels by riboflavin as photoinitiator. Int. J. Biol. Macromol..

[57] Hong B.M., Park S.A., Park W.H. (2019). Effect of photoinitiator on chain degradation of hyaluronic acid. Biomater. Res..

[58] Hong B.M., Kim H.C., Jeong J.E., Park S.A., Park W.H. (2020). Visible-light-induced hyaluronate hydrogel for soft tissue fillers. Int. J. Biol. Macromol..

[59] Parker S.T., Domachuk P., Amsden J., Bressner J., Lewis J.A., Kaplan D.L., Omenetto F.G. (2009). Biocompatible Silk Printed Optical Waveguides. Adv. Mater..

[60] Applegate M.B., Partlow B.P., Coburn J., Marelli B., Pirie C., Pineda R., Kaplan D.L., Omenetto F.G. (2016). Photocrosslinking of Silk Fibroin Using Riboflavin for Ocular Prostheses. Adv. Mater..

[61] Piluso S., Flores Gomez D., Dokter I., Moreira Texeira L., Li Y., Leijten J., van Weeren R., Vermonden T., Karperien M., Malda J. (2020). Rapid and cytocompatible cell-laden silk hydrogel formation via riboflavin-mediated crosslinking. J. Mater. Chem. B.

[62] Monnier V.M., Sell D.R., Saxena A., Saxena P., Subramaniam R., Tessier F., Weiss M.F. (2002). Glycoxidative and Carbonyl Stress in Aging and Age-Related Diseases. Critical Reviews of Oxidative Stress and Aging.

[63] Park S.-H., Seo S.-Y., Lee H.-J., Na H.-N., Lee J.-W., Woo H.-D., Son T.-I. (2012). Preparation of Furfuryl-fish gelatin (F-f.gel) cured using visible-light and its application as an anti-adhesion agent. Macromol. Res..

[64] Kong M.S., Koh W.-G., Lee H.J. (2022). Controlled Release of Epidermal Growth Factor from Furfuryl-Gelatin Hydrogel Using in Situ Visible Light-Induced Crosslinking and Its Effects on Fibroblasts Proliferation and Migration. Gels.

[65] AnilKumar S., Allen S.C., Tasnim N., Akter T., Park S., Kumar A., Chattopadhyay M., Ito Y., Suggs L.J., Joddar B. (2019). The applicability of furfuryl-gelatin as a novel bioink for tissue engineering applications. J. Biomed. Mater. Res. Part B Appl. Biomater..

[66] Nagiah N., El Khoury R., Othman M.H., Akimoto J., Ito Y., Roberson D.A., Joddar B. (2022). Development and Characterization of Furfuryl-Gelatin Electrospun Scaffolds for Cardiac Tissue Engineering. ACS Omega.

[67] Heo Y., Akimoto J., Kobatake E., Ito Y. (2020). Gelation and release behavior of visible light-curable alginate. Polym. J..

[68] Noh S.-H., Kim S.-W., Kim J.-W., Lee T.-H., Nah J.-W., Lee Y.-G., Kim M.-K., Ito Y., Son T.-I. (2019). Preparation of drug-immobilized anti-adhesion agent using visible light-curable alginate derivative containing furfuryl group. Int. J. Biol. Macromol..

[69] Donnelly P.E., Chen T., Finch A., Brial C., Maher S.A., Torzilli P.A. (2017). Photocrosslinked tyramine-substituted hyaluronate hydrogels with tunable mechanical properties improve immediate tissue-hydrogel interfacial strength in articular cartilage. J. Biomater. Sci. Polym. Ed..

[70] Han G.-D., Kim J.-W., Noh S.-H., Kim S.-W., Jang E.-C., Nah J.-W., Lee Y.-G., Kim M.-K., Ito Y., Son T.-I. (2019). Potent anti-adhesion agent using a drug-eluting visible-light curable hyaluronic acid derivative. J. Ind. Eng. Chem..

[71] Tong X., Pan W., Su T., Zhang M., Dong W., Qi X. (2020). Recent advances in natural polymer-based drug delivery systems. React. Funct. Polym..

[72] Nguyen A.K., Gittard S.D., Koroleva A., Schlie S., Gaidukeviciute A., Chichkov B.N., Narayan R.J. (2013). Two-photon polymerization of polyethylene glycol diacrylate scaffolds with riboflavin and triethanolamine used as a water-soluble photoinitiator. Regen. Med..

[73] Kim S.-H., Chu C.-C. (2009). Fabrication of a biodegradable polysaccharide hydrogel with riboflavin, vitamin B2, as a photo-initiator and L-arginine as coinitiator upon UV irradiation. J. Biomed. Mater. Res. Part B Appl. Biomater..

